# A Comprehensive Review on Iron-Based Sulfate Cathodes for Sodium-Ion Batteries

**DOI:** 10.3390/nano14231915

**Published:** 2024-11-28

**Authors:** Yalong Zheng, Zhen Zhang, Xinyu Jiang, Yan Zhao, Yichao Luo, Yaru Wang, Zhoulu Wang, Yi Zhang, Xiang Liu, Baizeng Fang

**Affiliations:** 1 School of Energy Sciences and Engineering, Nanjing Tech University, Nanjing 211816, China; yalongyun@163.com (Y.Z.); jiangxinyu@njtech.edu.cn (X.J.); joyeah1016@163.com (Y.Z.); ycluo2023@njtech.edu.cn (Y.L.); 202361208036@njtech.edu.cn (Y.W.); zhangy@njtech.edu.cn (Y.Z.); iamxliu@njtech.edu.cn (X.L.); 2 School of Chemistry and Molecular Engineering, Nanjing Tech University, Nanjing 211816, China; zhangzhen202109@163.com; 3 School of Chemical Engineering and Energy Technology, Dongguan University of Technology, Dongguan 523808, China

**Keywords:** SIBs, polyanionic compounds, sodium iron sulfate, electrochemical properties

## Abstract

Sodium-ion batteries (SIBs) are advantageous for large-scale energy storage due to the plentiful and ubiquitous nature of sodium resources, coupled with their lower cost relative to alternative technologies. To expedite the market adoption of SIBs, enhancing the energy density of SIBs is essential. Raising the operational voltage of the SIBs cathode is regarded as an effective strategy for achieving this goal, but it requires stable high-voltage cathode materials. Sodium iron sulfate (NFSO) is considered to be a promising cathode material due to its stable framework, adjustable structure, operational safety, and the high electronegativity of SO^4−^. This paper reviews the research progress of NFSO, discusses its structure and sodium storage mechanism on this basis, and summarizes the advantages and disadvantages of NFSO cathode materials. This study also evaluates the advancements in enhancing the electrochemical characteristics and structural reliability of SIBs, drawing on both domestic and international research. The findings of this paper offer valuable insights into the engineering and innovation of robust and viable SIB cathodes based on NFSO at ambient temperatures, contributing to their commercial viability.

## 1. Introduction

**Figure 1:**
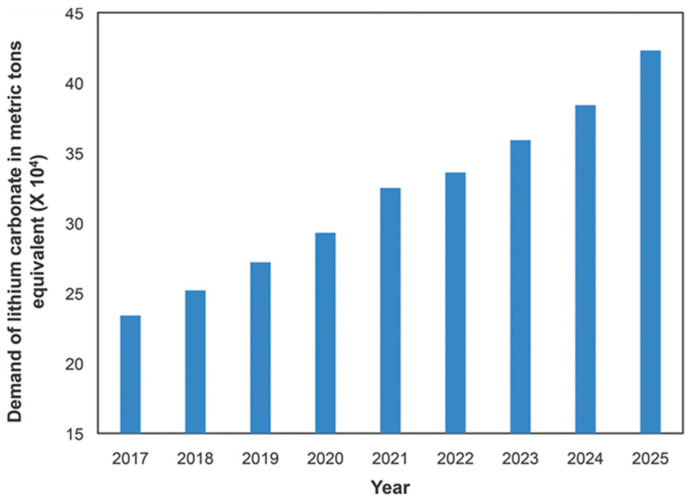
Demand for lithium in lithium carbonate equivalent. Ref. [15] Copyright 2019 Taylor and Francis Ltd.

In the wake of worldwide large-scale industrial growth over the past few decades, there has been a swift drawdown on non-renewable energy sources such as coal, oil, and natural gas. This has precipitated a range of critical concerns, encompassing the depletion of these finite resources, ecological harm, the exacerbation of the greenhouse effect, and the escalation of severe pollution [1,2,3]. The protection of the environment and the development of renewable energy are urgently needed [4,5,6,7,8,9]. Nonetheless, clean and renewable energy sources like solar, wind, geothermal, and tidal power face constraints due to their geographical specificity and susceptibility to weather variations. These factors introduce elements of intermittency and instability, which hinder their capability to fully tackle the pressing energy challenges of the present. Large-scale energy storage is a key technology to solve these problems [10,11]. Among various energy storage methods, electrochemical energy storage is the most convenient and effective. In recent decades, with the widespread attention and rapid development of LIBs, they have achieved great commercial success. However, lithium-ion batteries consume a large amount of lithium resources, further reducing the already scarce lithium ore resources and causing the cost of lithium batteries to rise [12,13,14]. As shown in Figure 1 [15], the demand for lithium has risen sharply, and countries have implemented trade protection for lithium resources. This implies that there is a pressing and urgent need to secure alternative resources from the Earth’s crust to supplement lithium reserves, given that the current lithium reserves are insufficient to satisfy the substantial requirements for energy storage. This is especially the case with the burgeoning demand for all-electric vehicles and plug-in hybrid electric vehicles, which are driving up the need for efficient energy storage solutions.

## 2. Overview of SIBs

### 2.1. The Structure of SIBs

**Figure 2:**
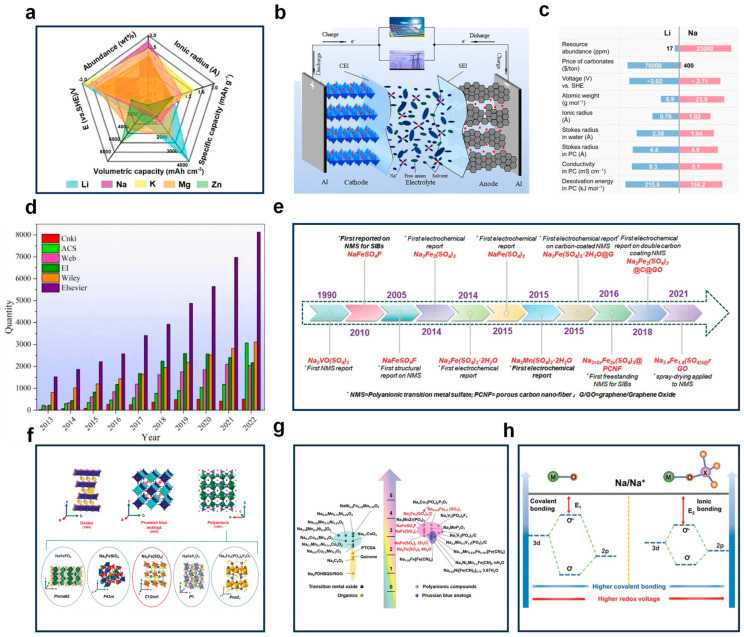
(**a**) The comparison illustrates the prevalence of resources in the Earth’s crust, the size of ionic radii, and the energy capacities—both in terms of weight and volume—along with the standard electrode potentials (referenced against the standard hydrogen electrode, SHE) for lithium, sodium, potassium, magnesium, and zinc when used as charge carriers in secondary batteries. Ref. [16] Copyright 2021 Wiley-VCH Verlag. (**b**) A schematic representation of the operating principle of sodium-ion batteries. Ref. [23] Copyright 2024 Elsevier Ltd. (**c**) A juxtaposition of the physical and chemical properties of sodium and lithium. Ref. [24] Copyright 2023 Wiley-VCH Verlag. (**d**) A statistical overview of academic publications related to sodium-ion batteries in leading databases over the past decade. Ref. [23] Copyright 2024 Elsevier Ltd. (**e**) A chronological outline highlighting significant advancements in the field of PSMs for SIBs. (**f**) An overview of the three predominant types of cathode materials proposed over the past four decades: transition metal oxides (TMOs), Prussian blue analogs (PBAs), and polyanionic compounds (**top**). Polyanions are further differentiated based on their constituent anionic functional groups into phosphates, silicates, sulfates, pyrophosphates, and mixed pyrophosphates (**bottom**). (**g**) A comparative analysis of the operational potentials in relation to Na/Na for a variety of cathode materials utilized in SIBs. (**h**) A diagrammatic representation of the electronic band structure for an oxide-based compound (**left**) and a polyanionic compound (**right**), demonstrating the polyanion’s effect on the ionicity of the M-O bond. Ref. [16] Copyright 2021 Wiley-VCH Verlag.

In an effort to partially replace lithium-ion batteries (LIBs), various alkali metal-ion batteries, including those based on sodium (Na^+^), potassium (K^+^), magnesium (Mg^2+^), and zinc (Zn^2+^), have garnered attention. Figure 2a presents a comparison of the performance characteristics of these alkali metal elements across different metrics [16]. Sodium-ion batteries (SIBs) emerge as top contenders for energy storage systems because of their favorable electrochemical performance characteristics, which closely resembles that of LIBs, coupled with the fact that the Earth’s abundance of sodium (2.83 wt%) significantly exceeds that of lithium (0.0065 wt%). This abundance not only makes SIBs a more sustainable option but also mitigates the supply chain concerns associated with lithium resources [17,18].

SIBs can be primarily divided into five components: the cathode, anode, separator, electrolyte, and aluminum current collector. The separator serves to isolate the cathode and anode, preventing short circuits that could occur from their contact. At the same time, they enable the unimpeded migration of sodium ions, acting as carriers that move back and forth between the cathode and anode, thereby enabling the processes of energy storage and release. SIBs utilize low-cost aluminum foil as the current collector; since sodium does not react with aluminum, the aluminum foil can be used for both the cathode and anode current collectors [19,20].

Similar to lithium-ion batteries (LIBs), SIBs operate on a rocking-chair mechanism [21,22]. As depicted in Figure 2b [23], during the discharge phase, sodium ions migrate from the anode to the cathode for storage, and during the charging phase, they are transported from the cathode back to the anode. Therefore, both cathode and anode materials must be capable of sodium storage reactions, with a potential difference between them. An increased potential difference is beneficial for enhancing the battery’s charge and discharge capacity.

### 2.2. Advantages of SIBs

As depicted in Figure 2c [24], sodium salts are more prevalent in the Earth’s crust and exhibit a broader distribution compared to lithium salts. Furthermore, SIB cathodes could potentially minimize the dependence on scarce metals like nickel (Ni) and cobalt (Co), leading to a substantial reduction in the overall cost of SIBs [25,26]. Furthermore, sodium does not alloy with aluminum at room temperature, which permits the utilization of more cost-effective and lighter aluminum in place of copper for the anode current collector. This further reduces costs and lightens the weight of the current collector. SIBs are safer compared to LIBs, with much less thermal runaway during the decomposition of electrodes and electrolytes [27,28]. Despite the sodium ion (Na^+^) being larger than the lithium ion (Li^+^), it exhibits a higher migration velocity within the electrolyte. This is attributed to its lower polarization and solvation energy. The reduced desolvation energy also aids in maintaining better capacity retention at reduced temperatures [29]. Sodium metal, being softer than lithium, can have its dendritic growth mitigated either through the application of mechanical pressure or by employing separators that possess a higher shear modulus [30,31].

### 2.3. SIBs Cathode Materials

Research on SIBs began in the 1970s, but the journey toward commercialization has been fraught with challenges and has progressed more slowly compared to that of LIBs. However, as shown in Figure 2d, research on SIBs has flourished in the past decade. In SIBs, the cathode material’s electrochemical attributes are pivotal in determining the battery’s overall performance. The theoretical energy density of the cathode material represents the upper limit of the battery cell’s energy density. The cathode material influences the power density of SIBs by affecting the capacity to accommodate sodium ions and the smoothness of the transport channels [32,33]. Additionally, the degradation of active material and the presence of impurities within the cathode can significantly influence the overall electrochemical performance of the battery. Therefore, the research on cathode sulfate materials, which is the main focus of this paper, has also developed rapidly, as shown in Figure 2e. The cathode materials for SIBs are commonly categorized into three main groups, as depicted in Figure 2f: transition metal oxides (TMOs) [34,35,36,37], Prussian blue analogs (PBAs) [38,39,40,41], and polyanionic compounds [42,43,44,45,46]. Layered transition metal oxides with large interstitial spaces for sodium storage are structurally simple, easy to synthesize, have high reversible capacity, offer high energy density and excellent rate capability, and are widely regarded as the most promising cathode materials for sodium ions. However, this stratified architecture is susceptible to structural degradation when it comes to accommodating the sizable Na ions during the insertion and extraction processes, which can result in a suboptimal cycle life. Additionally, the majority of layered oxides exhibit sensitivity to airborne moisture, complicating their storage [47,48,49]. Prussian blue and its analogs (PBAs) boast benefits such as cost-effectiveness, favorable rate capabilities, and tunable operating voltages. Yet, the inherent lattice water within them is challenging to eliminate, which undermines the chemical and structural integrity of the PBA materials. The heat generated during battery operation can cause the material to decompose, and the crystalline water formed during the material production process may damage the lattice structure, leading to safety issues. Polyanionic compounds (PSMs) can generally be represented in the form of A_x_M_y_ [(XO_m_)^n−^]_z_, where A is Li or Na, M is a variable valence metal ion, X represents elements such as phosphorus (P), sulfur (S), vanadium (V), silicon (Si), and so on. Polyanionic compounds are noted for their high operating voltage and excellent thermal stability. These materials possess a three-dimensional framework structure that typically undergoes minimal volume change during the extraction and insertion of sodium ions. Consequently, polyanionic cathode materials exhibit superior cycle stability and safety performance [50,51]. However, the inherent structure of these materials results in inferior electronic conductivity, which in turn leads to diminished specific capacity and subpar rate capability [52]. This paper mainly introduces the more promising cathode sulfate material NFSO, as shown in Figure 2g; among all the iron-based cathode materials reported in SIBs, it leads in terms of voltage. As shown in Figure 2h, the strong induction effect of the sulfate group can regulate the transition metal ions, helping to stabilize the lattice and limit the release of oxygen [16]. However, the sulfate family has hygroscopicity and low thermal stability; these materials are prone to decomposition at elevated temperatures, which restricts their synthesis and storage processes.

## 3. NFSO Cathode

### 3.1. Structure and Sodium Storage Mechanism

**Figure 3:**
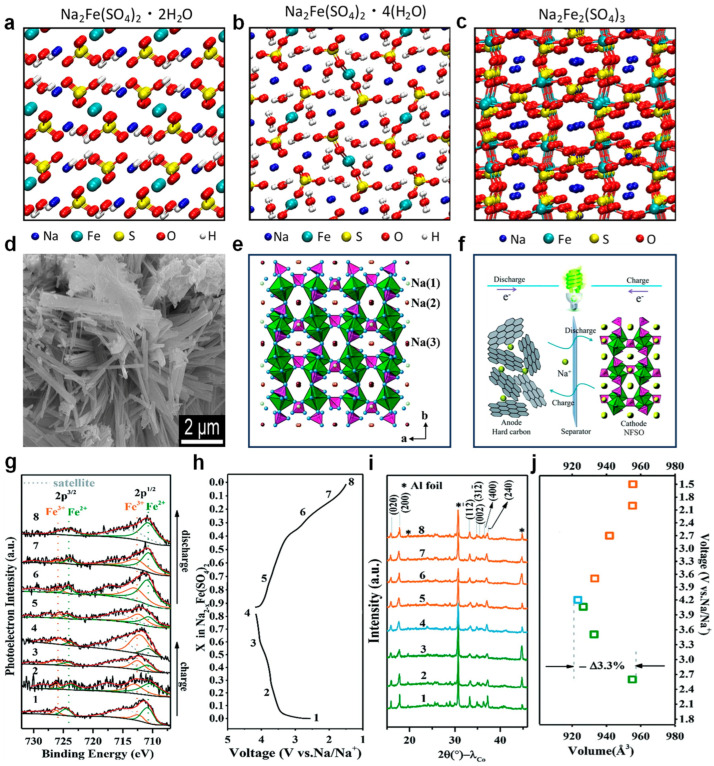
(**a**) The refined structure of Na_2_Fe(SO_4_)_2_·2(H_2_O) is presented. (**b**) The refined structure of Na_2_Fe(SO_4_)_2_·4(H_2_O) is depicted. (**c**) The refined structure of Na_2_Fe_2_(SO_4_)_3_, showcasing its architectural composition. Ref. [54] Copyright 2017 American Chemical Society. (**d**) Scanning electron microscopy (SEM) images of Na_2_Fe_2_(SO_4_)_3_ are displayed, providing a detailed view of the material’s microstructure. Ref. [56] Copyright 2019 Royal Society of Chemistry. (**e**) A schematic representation of the Na_2_Fe(SO_4_)_2_ structure along the c-axis is given, with FeO6 octahedra highlighted in green, SO_4_ tetrahedra in pink, and O atoms in blue. The Na(1), Na(2), and Na(3) atoms are distinguished by yellow, orange, and red colors, respectively. (**f**) An illustrative schematic of a complete cell configuration is shown, featuring NFSO/C as the cathode material and hard carbon as the anode. Ref. [57] Copyright 2019 Royal Society of Chemistry. (**g**) XPS Fe 2p narrow spectra of NFSO under different voltage states. (**h**) X in Na_2−x_Fe(SO_4_)_2_/C voltage curve under 0.1C. (**i**) Ex situ XRD patterns of NFSO/C at different charge/discharge states. (**j**) Cell volumes at corresponding potentials [57]. Copyright 2019 RSC.

“Structure determines performance” is an unassailable truth in materials science [53]. To understand the intrinsic properties and reaction mechanisms of electrode materials and to design high-rate cathode materials, grasping the structure of the material itself is fundamental. As illustrated in Figure 3a–c [54], NFSO exhibits a layered structure, within which Na^+^ and Fe^2+^ ions are situated at the octahedral sites of the crystal lattice. These octahedra are bridged by (SO_4_)^2−^ polyanions, a structural feature that endows NFSO material with good ionic conductivity, providing channels for the movement of sodium ions [54,55]. For example, Na_2_Fe_2_(SO_4_)_3_ crystallizes in a monoclinic system belonging to the P21/c space group and features a three-dimensional (3D) framework structure with sodium ion (Na^+^) diffusion pathways oriented along the c-axis. In this structure, FeO_6_ octahedra link together to create isolated Fe_2_O_10_ dimers through edge-sharing, which are further connected to SO_4_ tetrahedra at their vertices. This arrangement constructs a 3D framework that encompasses more spacious channels extending along the c-axis. The SEM image depicted in Figure 3d illustrates the morphology of the Na_2_Fe_2_(SO_4_)_3_ material [56]. Figure 3e delineates three distinct sites for Na^+^ within the structure, with Na2 and Na3 sites characterized by one-dimensional Na^+^ migration channels that run parallel to the c-axis. Figure 3f–j explains the sodium storage mechanism of NFSO when used as a cathode in SIBs. During discharge, Na^+^ migrates from the electrolyte to the surface of the cathode material and is inserted into the interlayer structure of NFSO under the influence of an electric field, while Fe^3+^ undergoes a reduction reaction to form Fe^2+^, a process known as the intercalation reaction [57]. During charging, Na^+^ migrates out of the crystal structure of sodium iron sulfate into the electrolyte, and Fe^2+^ is oxidized to Fe^3+^, a process known as the deintercalation reaction [57]. Na^+^ is stored and released in the crystal structure of NFSO through ionic diffusion, which refers to the migration of Na^+^ through channels such as vacancies and lattice defects within the crystal structure [58].

### 3.2. Advantages and Disadvantages of NFSO Cathode

#### 3.2.1. Advantages

(1) High Voltage Platform

**Figure 4:**
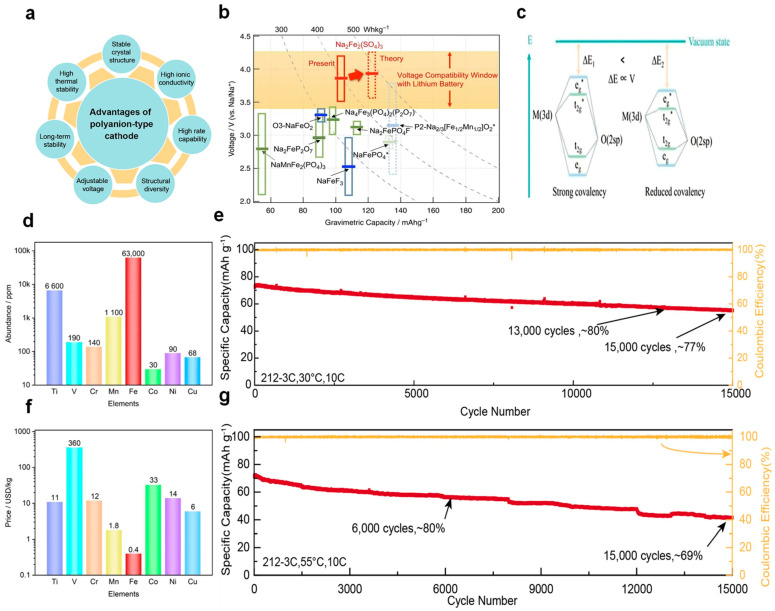
(**a**) The benefits of polyanion-type cathode materials are outlined, highlighting their unique properties and potential. Ref. [60] Copyright 2023 Elsevier. (**b**) A visual representation categorizes polyanionic cathode materials within green boxes and simple oxides/fluorides within blue, with horizontal bars indicating the average voltage. The yellow band signifies the voltage range that ensures compatibility with lithium-ion batteries. The novel compound Na_2_Fe_2_(SO_4_)_3_ is introduced with a red box and a theoretical capacity indicated by a dashed-red region. Ref. [60] Copyright 2014 Springer Nature. (**c**) A schematic illustration depicts the transformation of molecular orbitals, providing insight into the underlying electronic structure changes. Ref. [61] Copyright 2024 American Chemical Society. (**d**) A graphical representation of the elemental abundance of transition metals in the Earth’s crust is presented, emphasizing the availability of these elements. Ref. [62] Copyright 2023 MDPI. (**e**) The long-term cycling performance of the NFS_212_-3C cathodes at 30 °C is detailed, showcasing the endurance and stability of the material under specified conditions. Ref. [65] Copyright 2024 Elsevier. (**f**) The elemental price of transition metals is illustrated, indicating the economic considerations in material selection and development. Ref. [60] Copyright 2023 MDPI. (**g**) The long-term cycling performance of the NFS_212_-3C cathodes at an elevated temperature of 55 °C is presented, demonstrating the material’s performance under higher thermal conditions. Ref. [65] Copyright 2024 Elsevier.

Figure 4a illustrates the benefits of polyanionic materials [59], and compared to other polyanionic materials, iron-based sulfate polyanionic materials have been proven to enhance the operating voltage, making them a promising material. Initially presented by Yamada’s research group in 2014 as a cathode material for SIBs with a potential of 3.8 V (relative to Na/Na^+^), it was subsequently identified to possess a theoretical energy density reaching up to 540 Wh kg^−1^, coupled with a reversible specific capacity of 102 mAh g^−1^, as depicted in Figure 4b [60]. The high redox potential is attributed to its unique mesomeric effect, as shown in Figure 4c. In accordance with molecular orbital theory, the sharing of electrons between iron (Fe) and oxygen (O) results in the splitting of molecular orbitals into bonding and antibonding types. As the covalent bond between Fe and O strengthens, the energy gap between these antibonding and bonding orbitals widens. Electrons preferentially occupy the lower-energy bonding orbitals, which in turn increases the population of electrons in the antibonding orbitals, thereby diminishing the energy gap (Δ) between the antibonding orbitals and the vacuum level, resulting in a decreased redox potential. Conversely, the introduction of a sulfur (S) atom, which is more electronegative, to create an Fe-O-S linkage, weakens the Fe-O covalent bond, thereby increasing the Δ value; this results in a higher voltage, which in turn enhances the redox potential [61].

(2) Cost-Effectiveness

The components of sodium iron sulfate are notably cost-effective, with iron being particularly abundant, holding the fourth position in terms of abundance in the Earth’s crust and known for its plentiful reserves. Figure 4d and Figure 4f, respectively, depict the prevalence of various transition metals within the Earth’s crust and the pricing of these transition metals [62]. Sodium resources are also very rich and widely distributed, which also means that the material supply chain is more stable on a large scale, helping to improve the efficiency and reliability of the entire battery system. Common recycling methods currently include material regeneration and reducing hydration issues. Material regeneration involves regenerating NFSO materials through methods such as solvent thermal treatment and mechanical grinding for further use. Reducing hydration issues is achieved by combining carbon-based materials or using encapsulation techniques, which can minimize NFSO’s sensitivity to moisture, thereby improving its recyclability. In the future, closed-loop recycling systems can be developed to ensure the continued availability of materials while minimizing environmental impact [63,64,65].

(3) Good Safety

NFSO demonstrates high chemical stability, which makes it less susceptible to side reactions during the battery’s charge and discharge cycles. This stability helps to reduce chemical changes within the battery, thereby lowering the risk of thermal runaway or other safety issues. Secondly, compared to other battery materials containing toxic heavy metals, NFSO does not contain harmful elements, and even in the event of battery damage or leakage, its impact on the environment and human health is minimal [66,67].

(4) Stable Cycling

Performance NFSO exhibits good cycling stability in sodium-ion batteries, maintaining high coulombic efficiency and capacity retention even after a high number of cycles. This is because NFSO undergoes very small volume changes during the Na^+^ insertion/deinsertion process, less than 5%, ensuring good structural stability over long-term cycling [68]. The Na_2_Fe(SO_4_)_2_ synthesized by Zheng and others maintained capacity retention rates of 77% and 69% after 15,000 cycles at a 10 C rate at 30 °C and 55 °C, respectively, as shown in Figure 4e,g [65]. Note that the structural stability is also related to the H_2_O units in the structure, for example, the absence of H_2_O units leads to different phase transition reactions between Na_2_Fe(SO_4_)_2_ and Na_2_Fe(SO_4_)_2_·2H_2_O.

#### 3.2.2. Disadvantages

(1) Slow Kinetics

**Figure 5:**
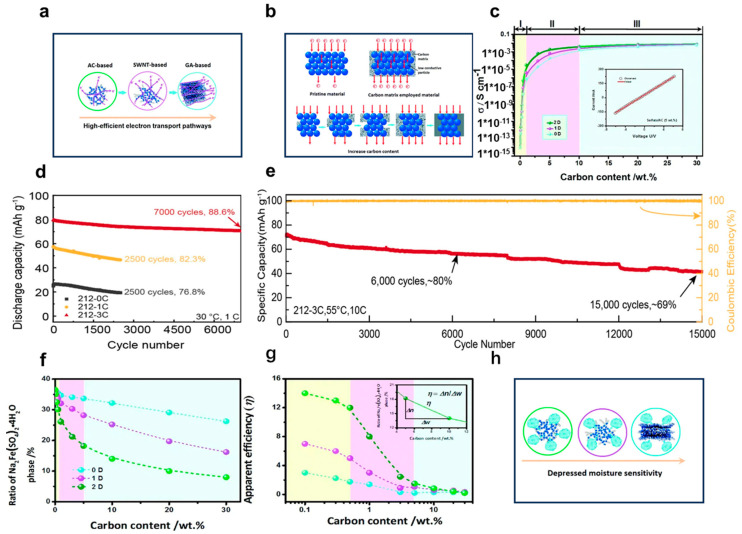
(**a**) Diagrammatic representation of the electron transfer routes within composites fabricated from activated carbon (0D), single-walled nanotubes (1D), and graphene (2D). (**b**) Illustration of electron transfer mechanisms in materials with poor conductivity (left side) and those enhanced by a carbon matrix (right side). The correlation between the capacity for electron transfer and the concentration of carbon in the matrix is depicted. (**c**) Comparative analysis of the electronic conductivity for sulfate/C composites as recorded in the materials repository. A demonstration of the linear regression on the current-voltage graph for the sulfate/AC composite with 5 wt% carbon content is presented as an inset within (**c**). (**d**) Cycle performance of the NFS cathodes at 1 C. (**e**) are long-term cycle performance of the NFS212-3C cathodes at 55 °C [65]. Copyright 2024 Elsevier. (**f**) Chart showing the fluctuations in the content of hydration products. (**g**) Graphical depiction of the changes in the apparent efficiency for the sulfate/C composite across various structural configurations. (**h**) Outline of the hydration mechanisms observed in composites based on activated carbon, carbon nanotubes, and graphene. Ref. [76] Copyright 2016 Royal Society of Chemistry.

Despite its numerous advantages, NFSO is hindered by its low electronic conductivity, a limitation that is associated with the electron interactions within the (SO_4_)^2−^ anionic groups during the electrochemical reaction. The transition metal iron ions are frequently insulated by the polyanionic (SO_4_)^2−^ groups, which are not conducive to electron transfer. The valence electron cloud is isolated, hindering electron exchange, resulting in an extremely low intrinsic electronic conductivity of the material, which limits the practical application of NFSO cathode. For cathode materials to achieve high-rate charging and discharging, it is necessary for sodium ions within the structural framework to diffuse smoothly through channels and for electrons to quickly transfer through the electrode material and interface to the external circuit. If either ion or electron conduction is impaired within the active material of the cathode, this limitation can restrict the overall rate capability of the electrode material and may even have repercussions on the total power output of the sodium-ion battery (SIB) [69]. Compared to lithium ions, sodium ions have a slower insertion rate in NFSO, which affects the battery’s charging and discharging performance, especially under high current demands. Consequently, numerous studies are focused on enhancing conductivity by employing diverse approaches like applying a carbon layer [70,71,72,73], decreasing particle dimensions, and crafting ideal shapes [74,75]. Meng et al. carried out a comprehensive investigation into the correlation between the structural composition and the physicochemical characteristics of NFSO/C composites. This was achieved by utilizing three distinct types of carbon frameworks, activated carbon (AC: 0-dimensional), single-walled carbon nanotubes (CNT: 1-dimensional), and graphene (GA: 2-dimensional), to fabricate composites with varying structural configurations of sulfate/carbon, as shown in Figure 5a. The structure of these composites formed with NFSO is displayed. Figure 5b clearly illustrates the substantial influence of the carbon matrix on electronic conductivity; prior to reaching a certain proportion of the composited carbon matrix, the electronic conductivity of the composite progressively is enhanced with the rising content of carbon. Once a certain threshold is attained, the electronic conductivity plateaus and does not escalate further with additional increments in carbon content. The trend is similarly conveyed by the curve depicted in Figure 5c [76].

(2) Interface Instability

At high voltages, the interface stability between NFSO and the electrolyte is a challenge. NFSO might absorb moisture, leading to the formation of hydrates or other chemical changes on its surface; not only does this involve the intrinsic properties of the cathode material itself, but also the interactions between the cathode material and the electrolyte during the battery’s charge and discharge cycles, which can potentially result in the development of an unstable solid electrolyte interphase (SEI). These interface reactions could consume the electrolyte and produce gas, affecting the battery’s stability and energy density. Improvements through interface engineering or other strategies are needed [77].

Zheng et al. successfully combined carbon nanotubes (CNTs) with Na_2_Fe(SO_4_) using a well-designed mechanochemical method. The results showed that Na_2_Fe(SO_4_)·4H_2_O is an intermediate phase formed during the mechanochemical reaction. After mild thermal treatment, Na_2_Fe(SO_4_)·4H_2_O undergoes dehydration to produce the Na_2_Fe(SO_4_)_2_ cathode material. As shown in Figure 5d,e, the composite material retains a capacity of 70.5 mAh g⁻¹ after 6974 cycles at a current density of 1C. At 55 °C and a current density of 10C, the capacity retention is as high as 80.1% after 12,814 cycles, demonstrating exceptional durability. The outstanding cycling performance is attributed to the minimal lattice changes during Na-ion extraction/insertion and the permeating CNT network [65].

(3) Thermal Reduction and Hygroscopicity

The material’s sulfate radicals are typically unstable at temperatures exceeding 450 °C, tending to emit sulfur dioxide (SO_2_) gas, which may lead to safety concerns in real-world applications. Additionally, due to the hygroscopic nature of the sulfate radicals, it is imperative that NFSO materials are synthesized and stored in a moisture-free setting. The formation of hydrates can compromise the original crystalline structure of the sulfate cathode and its associated electrochemical properties. Furthermore, the water absorbed by the sulfate may intensify side reactions between the electrolyte and the electrode, resulting in irreversible loss of capacity. Meng et al. A methodical analysis was performed to explore the link between the structural makeup and the physicochemical traits of sodium iron sulfate/carbon (NFSO/C) composites. This was achieved by utilizing three distinct carbon substrates, activated carbon (AC: zero-dimensional), single-walled carbon nanotubes (CNT: one-dimensional), and graphene (GA: two-dimensional), to fabricate composites with varied sulfate/carbon configurations. The data presented in Figure 5f encapsulates the proportion of hydrated products for the aforementioned samples following a 240-hour exposure period to ambient air. Relative to the pristine material, there is a notable reduction in the hydrated phase content within the sulfate/carbon composites, which suggests that the integration of a carbon substrate can mitigate the material’s susceptibility to moisture. The observed trend in hydrate product ratios, with 0D (AC) exceeding 1D (CNT), which in turn exceeds 2D (GA), implies that encapsulation is a more effective strategy for curbing hydrate formation. Figure 5g delineates the trend of apparent efficiency in response to escalating carbon substrate quantities. During the initial phase characterized by a low carbon substrate concentration (Phase I: 0.1–1 weight percent), the hydrated phase of the materials experiences a swift decline as carbon substrate content escalates, while the apparent utilization efficiency of the carbon substrate descends at a more moderate pace. The findings indicate that a modest amount of carbon substrate incorporation exerts a beneficial suppressive effect on the hydration reaction. At a constant carbon substrate content, the GA-based composite material demonstrates superior apparent efficiency and a minimal hydrated phase, in contrast to the AC-based composite material, which exhibits the opposite characteristics. These results underscore that the GA-based composite material is less prone to moisture, whereas the AC-based composite material is more moisture-sensitive. This disparity is attributed to the differing structural architectures, as depicted in Figure 5h, where the GA-based composite material possesses a compact sandwich-like structure that affords robust protection to the sulfate, thereby curtailing hydration reactions effectively. In contrast, the AC-based composite material, with its loose structure, expansive surface area, and pronounced porosity, fails to provide adequate protection to the sulfate, resulting in heightened moisture absorption. In the subsequent phase (Phase II: 1–5 weight percent), as carbon substrate content continues to increase, the apparent efficiency of the composite materials experiences a rapid decline. In extreme scenarios, during the final phase (Phase III: 5–30 weight percent), the apparent efficiencies for all composite materials reach a plateau, stabilizing at their minimum values. The findings suggest that only a relatively low concentration of carbon substrate doping can effectively mitigate moisture sensitivity. Although an increase in carbon substrate content can diminish the formation of hydration products, this is counterbalanced by a reduction in utilization efficiency. When an excessive amount of carbon substrate is incorporated, its impact on the formation of hydration products becomes negligible. This leads to a very low carbon doping efficiency (close to zero). Therefore, the effective areas to suppress the moisture sensitivity of sulfate are Region I and Region II (≤5 wt%). Considering the low carbon utilization efficiency, when the carbon content in the sulfate/C composite exceeds 5 weight percent, entering Stage III, the impact on the material’s properties becomes marginal [76].

Although sodium iron sulfate (NFSO) offers advantages such as high voltage, cost-effectiveness, and safety, its practical application is hindered by issues including low electronic conductivity, interface instability at high voltages, and thermal reduction and hygroscopicity. To address the issue of low electronic conductivity, elements such as cobalt (Co), manganese (Mn), or fluorine (F) can be introduced through doping. These elements can alter the crystal structure of the material, thereby enhancing its electronic conductivity and stability. For instance, cobalt doping can accelerate reaction kinetics, while fluorine doping can adjust the redox potential, reduce the band gap, and improve conductivity. To mitigate interface instability, a carbon layer or polymer coating can be applied to the surface of NFSO, reducing direct contact between the active material and air or electrolytes and suppressing side reactions. Such coatings not only protect the material from moisture but also enhance the efficiency of sodium-ion transfer at the interface. Additionally, novel electrolytes, such as high-stability fluorides or borate-based electrolytes, can be developed. These electrolytes can minimize side reactions and form a stable solid electrolyte interphase (SEI) layer, protecting the electrodes. To address the challenges of thermal reduction and hygroscopicity, NFSO materials can be stored in an anhydrous environment or encapsulated using techniques such as hydrophobic coatings. Limiting exposure to moisture helps preserve the integrity of the material’s crystal structure, reducing the risk of electrochemical performance degradation.

### 3.3. Modification Methods

In addition to the inherent properties of materials, such as sodium ion diffusion coefficients, factors impacting the electrochemical performance also encompass the diffusion distance that sodium ions must travel and the electronic conductivity of the electrode materials. These involve issues related to the interface and surface of the overall electrode material, as well as more macroscopic material morphology design issues, which are currently the most extensively and comprehensively researched directions. To tackle these key issues, strategies to boost the electrochemical performance of electrode materials involve techniques such as surface modification and structural optimization [36,78,79,80]. Utilizing one or a combination of these approaches can frequently lead to a significant improvement in the materials’ electrochemical performance.

#### 3.3.1. Surface Modification

Surface modification refers to the creation of a protective layer on the surface of a material through either physical or chemical means. To enhance the structural stability of cathode materials, protective coatings have been developed to regulate sodium ion transfer at the interface and suppress side reactions to protect active materials. The most common method is carbon coating [81,82], which has the following advantages.

Applying a carbon coating can facilitate electron transfer among particles, thereby advantageous for augmenting the material’s rate capability. The carbon coating acts as a robust barrier against air and moisture, creating a reliable shield on the exposed material that guards the reactive components from degradation, simplifying its storage and transportation and thereby enhancing its potential for commercial application. The carbon layer serves the role of a reducing agent, preventing the oxidation of certain Fe^2+^ ions, thereby minimizing the deposition of impurities on the electrode surface. The carbon layer also inhibits particle aggregation and expansion during the synthesis process, effectively suppressing material volume expansion, which is conducive to optimizing material performance. For instance, the process of combining ascorbic acid with other precursor materials and sintering them in situ results in the formation of a carbon layer that serves to shield NFSO. The carbon layer exhibits exceptional resistance to corrosion and maintains stability when in contact with typical electrolytes. This property helps to mitigate unwanted side reactions that could occur between the electrolyte and NFSO materials, thereby enhancing the cyclic durability of NFSO electrodes.

**Figure 6:**
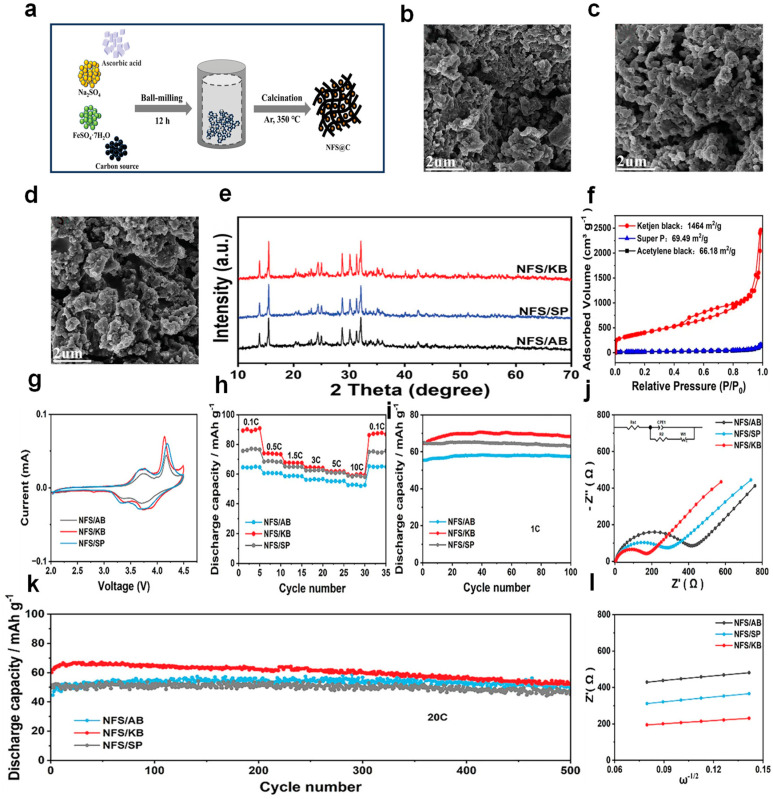
(**a**) A diagrammatic representation of the NFS/C synthesis process is provided. SEM images are displayed for (**b**) NFS/AB, (**c**) NFS/KB, and (**d**) NFS/SP, showcasing the morphological characteristics of these materials. (**e**) The X-ray diffraction (XRD) patterns for NFS/KB, NFS/SP, and NFS/AB are presented, indicating their crystallographic structures. (**f**) N_2_ adsorption and desorption isotherms for Acetylene black, Ketjen black, and Super P are depicted, illustrating their porous properties. (**g**) The first-cycle CV profiles at a scan rate of 0.1 mV s^−1^ for NFS/C are shown, reflecting the initial electrochemical behavior. (**h**) The rate capability testing results at various current rates are displayed, demonstrating the performance under different power demands. (**i**) The cycling stability at a current rate of 1 C is presented, showing the endurance of the material over repeated cycles. (**j**) Electrochemical Impedance Spectroscopy (EIS) analysis is conducted to understand the charge transfer and diffusion processes. (**k**) The cycling stability at a higher current rate of 20 C is illustrated, indicating the material’s performance under more demanding conditions. (**l**) The impedance versus frequency plots are provided, offering insights into the kinetic properties of the material. Ref. [83] Copyright 2024 Wiley-VCH Verlag.

In Deng et al.’s research, various types of conductive carbon materials were utilized as carbon precursors to fabricate a range of in situ carbon-coated NFSO (NFSO@C) composites through a straightforward solid-phase synthesis approach, as shown in Figure 6a. These carbon sources include acetylene black, Ketjen Black (KB), and Super P. Figure 6b–d show the SEM images of these three carbon sources and NFSO composites, and Figure 6e shows the XRD of the three composite materials, all having similar peak shapes. Subsequent research found that NFSO combined with KB (NFSO@KB) exhibited the largest specific surface area, as shown in Figure 6f, which is conducive to electrolyte penetration and rapid ion/electron migration, thereby improving electrochemical performance. Preliminary studies on the electrochemical performance of NFSO/C half-cells presented the initial cycle CV (Cyclic Voltammetry) curves for NFSO/AB, NFSO/KB, and NFSO/SP, as depicted in Figure 6g. Among the NFSO/C samples, NFSO/KB, due to its uniform and continuous conductive network structure, showed higher peak intensity, larger integral area, and smaller polarization. All samples based on the constructed dual-carbon structure showed excellent rate performance, as shown in Figure 6h. Notably, the NFSO/KB exhibited a reversible capacity of 92 mAh g^−1^ at a current rate of 0.1 C. Upon reverting to the 0.1 C rate following high-rate cycling tests, it maintained a reversible capacity of 90 mAh g^−1^. The endurance performance of NFSO/C under varying current rates was further evaluated to assess the material’s capacity to sustain its charge-discharge characteristics over time. Encouragingly, as depicted in Figure 6i, the results indicate the material’s robust cycling stability. NFSO/KB showed good cycling stability at 1 °C (Maintaining 95% of its initial capacity following 100 charge-discharge cycles), mainly because the NFSO nanoparticles were dispersed in the carbon network structure of KB, improving electron and ion conductivity. Subsequently, the three composite materials underwent testing, where NFSO@KB demonstrated an extended cycle life (exhibiting a capacity of 67 mA h g^−1^ after enduring 500 cycles at a rate of 20 °C, with an impressive capacity retention of 85%), as illustrated in Figure 6k. Finally, electrochemical impedance testing (EIS) was performed to preliminarily evaluate the conductivity and kinetic performance of the NFSO/C cathode half-cells, with the results shown in Figure 6j,l. Clearly, NFSO/KB demonstrated the lowest charge transfer resistance and diffusion resistance, primarily attributed to the in situ carbon coating, which enhanced the rapid transport pathways for electrons and ions, with uniform NFSO particles embedded in the continuous Ketjen black carbon network, which is more conducive to Na^+^ diffusion [83]. Yao et al. utilized a simple homogeneous strategy to combine Na_2_Fe(SO_4_)_2_ (NFSO) with graphene. Due to the nanoscale Na_2_Fe(SO_4_)_2_ being supported by a robust crosslinked carbon matrix composed of rGO sheets and carbon dots, electron transfer and reaction kinetics were effectively enhanced. The small rGO sheets act as collectors, significantly improving electron transport, suppressing aggregation, and ensuring smooth ion pathways [73].

Despite the many benefits of carbon coating, attention should be paid to the following: (a) An excessive application of carbon coating in the composite material does not enhance its electrochemical performance; it might actually lead to a decrease in the overall energy yield. Therefore, a thin and uniform layer of carbon coating is optimal for maximizing the energy density of SIBs. (b) This method is simple and cost-effective, but since the residual carbon is mostly amorphous and difficult to graphitize, there is still much room for improving electronic conductivity, and it is greatly influenced by the preparation conditions, which can easily lead to uneven coating and cracking during the cycling process. To address these issues, many studies have directly incorporated better-conducting carbons, such as nanoballs (0D), carbon nanotubes (1D), graphene, or other nanocarbon layers (2D), dispersed into the precursor and subjected to high-temperature sintering, which has also achieved certain results. Alternatively, a combination of the two methods can be used, compounding carbon-coated particles with a small amount of conductive carbon, which may achieve the goal of building a complete conductive network to improve electrode kinetic performance while also protecting the electrode and improving cycling performance. In addition to surface modification with carbon materials, The surfaces of SIB cathode materials can also be coated with conductive polymers, including but not limited to polypyrrole (PPy) [84,85], polythiophene (PTh) [86], and their derivatives such as poly(3,4-ethylenedioxythiophene) (PEDOT) [87,88].

#### 3.3.2. Structural Modification

Common approaches to structural design encompass nano-engineering techniques [89,90], hierarchical structure design [91,92,93], and elemental doping [94,95,96].

(1) Nano-Engineering

One of the contributing factors to the decline in capacity for cathode materials is their excessive size or dimensions. The larger the electrode particles, the more non-reactive parts there are, and the longer the diffusion distance for Na ions. Consequently, regulating the size of the active material to reduce the sodium ion diffusion pathway is a proven and prevalent strategy for enhancing electrochemical performance [89,97]. Nano-engineering can be simply understood as creating porous, flake-like morphologies or nanostructures of different dimensions, such as nanowires and nanorods, to increase the material’s surface area, further accelerating the transfer rate of electrons and ions at the interface and effectively reducing the Na^+^ diffusion distance. This enhancement has been validated through both computational modeling and experimental evidence. When the composite is reduced to the nanoscale, the contact area between the NFSO composite and the electrolyte expands, and nano-engineering, which is advantageous for revealing a greater number of active centers for redox reactions, contributes to superior electrochemical activity. A large surface area can enhance surface energy, but it may also lead to increased aggregation of NFSO materials, potentially diminishing the benefits of nano-engineering. The implementation of micro-nano structures can counteract the aggregation of NFSO, harnessing synergistic benefits and leveraging the performance improvements afforded by nanostructures, thereby resolving a range of challenges associated with nano-engineering.

For example, Nam et al. prepared sodium iron sulfate nanofibers using the electrospinning method. The synthesized material had a size of approximately 400–800 nm, and this structure was beneficial for reducing the diffusion distance of Na^+^. Furthermore, the authors applied a carbon coating on the surface of sodium iron sulfate using polyvinylpyrrolidone, which further enhanced the material’s electrical conductivity and improved ion transport performance [98].

(2) Hierarchical Structure Design

**Figure 7:**
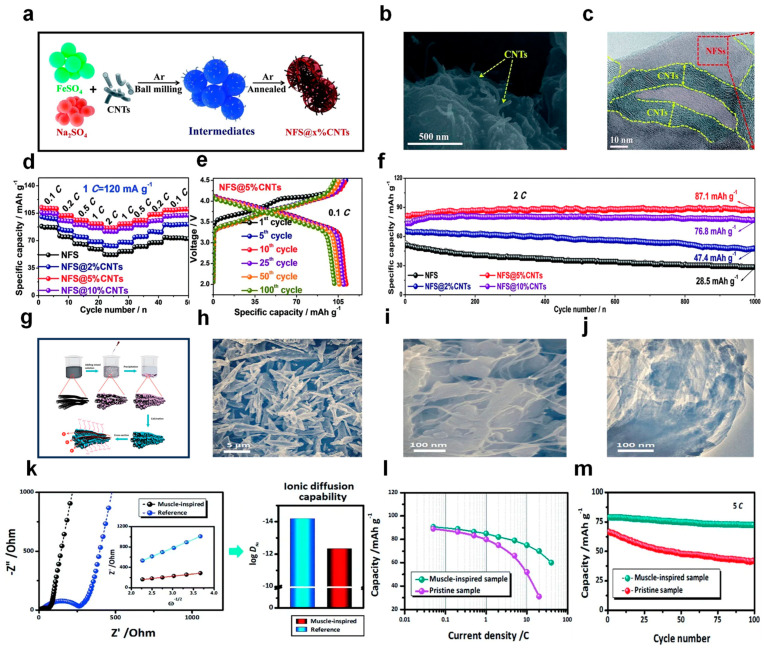
(**a**) A diagrammatic depiction of the fabrication methods for the NFS@x%CNTs nanocomposites. (**b**) A field emission scanning electron microscopy (FESEM) image and (**c**) a high-resolution transmission electron microscopy (HRTEM) image of the NFS@5%CNTs composite. (**d**) The rate capabilities of various NFS@x%CNTs cathode materials were evaluated at a range of C rates. (**e**) Voltage profiles for the NFS@5%CNTs during the initial, 5th, 10th, 25th, 50th, and 100th cycles at a 0.1 C rate. (**f**) Endurance cycling performance of the NFS@x%CNTs at a 2 C rate within a potential window of 2.0–4.5 V versus Na/Na^+^. Ref. [99] Copyright 2019 Royal Society of Chemistry. (**g**) A schematic of the top-down synthesis approach and the structure of the muscle-inspired Na_2+2x_Fe_2−x_(SO_4_)_3_/SWNT spindles, including the preparation process of the “top” hydrated precursor, the structure of the “down” alluaudite product, and its cross-sectional view featuring expedited electron and ion transport pathways. SEM images of the composite at (**h**) low and (**i**) high magnifications. (**j**) TEM images of the composite. (**k**) Impedance spectroscopy Nyquist plots and the linear correlation between Z′ and the inverse square root of frequency in the low-frequency region (inset), along with a comparison of the calculated sodium diffusion coefficients among the samples. (**l**) A comparison of the rate performance between the muscle-inspired spindle and the unmodified sample. (**m**) Durability cycling performance of the muscle-inspired spindle and the pristine sample at a 5 C rate [100] Copyright 2015 Royal Society of Chemistry.

Hierarchical structure design involves confining particles to nanoscale dimensions in at least one aspect, transcending the approach of mere simple blending. One-dimensional structures such as nanofibers and nanowires have the advantage of short Na^+^ diffusion distances. Meanwhile, graphene can be fashioned into a 3D network structure, which aids in bolstering the structural stability. Li et al. used a ball-milling method to easily introduce a CNT framework into the NFSO matrix to prepare NFSO@x%CNTs nanocomposites with a hierarchical structure, as shown in Figure 7a. In the composite material, the nanostructured CNTs penetrate the entire sodium iron sulfate framework, forming a conductive network woven by carbon nanofibers and particles. The physical appearance and structural aspects of the NFSO@5%CNTs composite were examined through field emission scanning electron microscopy (FESEM) and high-resolution transmission electron microscopy (HRTEM). The FESEM image in Figure 7b reveals numerous CNT terminations on the NFSO particle surfaces, reinforcing the notion that CNTs are dispersed within NFSO. The HRTEM image in Figure 7c illustrates the presence of various CNT conduits traversing the NFSO particle interiors, corroborating this structural integration. The influence of the integration of CNTs on the electrochemical performance of the NFSO@x%CNTs cathode materials was then thoroughly assessed and contrasted with the pristine NFSO material. Figure 7d juxtaposes the rate performances of the NFSO@x%CNTs cathodes under varying current densities, ranging from 0.1 C to 2 C, followed by a return to 0.1 C (1 C = 120 mA g^−1^). The unmodified NFS cathode exhibited a pronounced capacity fade, with an 84% retention, likely due to its suboptimal electronic conductivity and the absence of structural reinforcement from CNTs. Conversely, the NFSO@5%CNTs cathode material consistently demonstrated superior rate capability, delivering specific capacities of 110.2, 102.1, 96.3, 91.8, and 86.4 mA h g^−1^ at rates of 0.1, 0.2, 0.5, 1, and 2 C, respectively. Remarkably, upon reverting to 0.1 C after the high-rate electrochemical assessment, the NFSO@5%CNTs cathode material sustained a capacity retention of 98% (107.9 mA h g^−1^). This exceptional capacity maintenance signifies that the CNTs-enhanced NFSO cathode material possesses a resilient structure even amidst rapid sodium ion migration dynamics. The charge-discharge profiles of the NFSO@5%CNTs cathode material post-multiple cycles at 0.1 C are depicted in Figure 7e, with overlapping curves, which is indicative of enhanced material stability over cycles. As illustrated in Figure 7f, the refined NFSO@5%CNTs cathode material preserved an elevated specific capacity of 87.1 mAh g^−1^ after enduring 1000 cycles at 2 C, significantly superior to other materials. The superior cycling and structural stability can likely be credited to the intertwined carbon nanotube network, which mitigates volume fluctuations during the intercalation process [99]. Meng et al. synthesized NFSO/SWNT composites through a top-down synthetic method, as shown in Figure 7g. Figure 7h,i are SEM images of this composite, showing uniform spindle-shaped particles, and Figure 7j is a TEM image, Illustrating nanoparticles of Na_2+2x_Fe_2−x_(SO_4_)_3_ with diameters ranging from 50 to 100 nanometers, effectively enveloped by a layer of single-walled carbon nanotubes. Subsequently, Electrochemical Impedance Spectroscopy (EIS) was employed to evaluate the ion diffusion capability. Figure 7k indicates that the composite material exhibits reduced impedance, thereby possessing a higher sodium ion diffusion coefficient. Figure 7l shows that as the rate increases, the rate of decline of the composite material’s specific capacity is smaller than that of the raw material, i.e., it has better rate performance. Figure 7m is a cycling performance graph of the two materials. The composite material demonstrated a capacity retention rate of 92% following 100 cycles at a charging rate of 5 C, far higher than the raw material’s 64% [100].

Hierarchical structure design can not only be combined with carbon nanotubes but also with nano-flower-like hierarchical structures, which possess a larger surface area, thus facilitating the diffusion of sodium ions and enhancing the wettability of the electrolyte, which in turn leads to superior rate performance [101,102].

(3) Elemental Doping/Replacement

**Figure 8:**
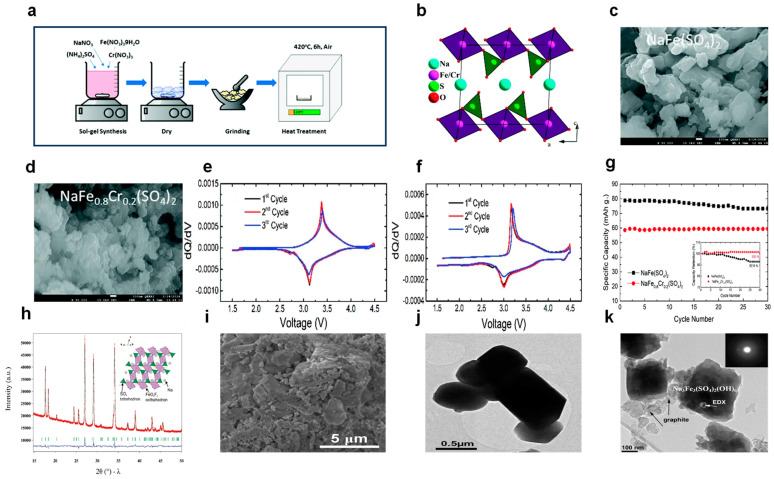
(**a**) A diagrammatic representation of the sol-gel synthesis procedure for the preparation of NaFe_x_Cr_1−X_(SO_4_)_2_, with varying chromium content (X = 0, 0.8, 1.0). (**b**) The crystallographic structure of NaFe_0.8_Cr_0.2_(SO_4_)_2_ is depicted in projection along the [010] direction. SEM micrographs are provided for (**c**) the undoped NaFe(SO_4_)_2_ and (**d**) the chromium-doped NaFe_0.8_Cr_0.2_(SO_4_)_2_, highlighting the refinement in particle size due to chromium incorporation. The differential capacity versus voltage (dQ/dV) plots are presented for (**e**) NaFe(SO_4_)_2_ and (**f**) the NaFe_0.8_Cr_0.2_(SO_4_)_2_, showcasing the electrochemical behavior. (**g**) The cyclic performance of both NaFe(SO_4_)_2_ and NaFe_0.8_Cr_0.2_(SO_4_)_2_ is compared, with an inset detailing the capacity retention percentage. Ref. [105] Copyright 2018 Royal Society of Chemistry. (**h**) The powder X-ray diffraction (XRD) patterns for NaFeSO_4_F are displayed. (**i**) SEM images of the solid-state synthesized NaFeSO_4_F are shown. (**j**) A transmission electron microscopy (TEM) image of NaFeSO_4_F is provided. Ref. [103] Copyright 2010 American Chemical Society. (**k**) A low magnification bright field TEM image of the completely reduced phase of Na_3_Fe_3_(SO_4_)_2_(OH)_6_ is displayed, along with the corresponding electron diffraction (ED) pattern. Ref. [109] Copyright 2015 Elsevier.

Elemental doping or substitution involves the deliberate introduction of a small quantity of other elements into a pure substance, which is essential for boosting electronic conductivity by modifying the structure of NFSO [103,104]. Surface coatings can enhance surface conductivity through the creation of conductive layers, and downsizing particles can shorten the pathways for ion and electron transfer. However, merely reducing particle size increases the contact area between the electrode and the electrolyte and does not inherently improve conductivity. Doping, on the other hand, is an effective method to augment the intrinsic conductivity, thus influencing the electrochemical performance. A variety of doping or substitution techniques have been employed to overcome the limitations inherent in NFSO. It has been proven that doping with Mn or Al enhances structural and thermal stability; Co doping increases reaction kinetics, and doping with Mg/Zr/Ti adjusts conductivity and lifespan. To achieve optimal battery performance, both the doping element and its concentration should be considered. There are two main methods to improve the material’s intrinsic electronic conductivity through doping: one is by introducing additional metal ions, where isovalent metal ions create metal polyhedron junction points. For example, Umair Nisar and his team created a series of NaFexCr_1−X_(SO_4_)_2_ compounds, where X equals 0, 0.8, and 1.0, through a sol-gel technique with the incorporation of chromium ions, as depicted in Figure 8a. The projection of the crystal lattice structure for NaFe_0.8_Cr_0.2_(SO_4_)_2_ along the [010] plane is presented in Figure 8b. Figure 8c and 8d display the scanning electron microscopy (SEM) images for NaFe(SO_4_)_2_ and the chromium-doped NaFe_0.8_Cr_0.2_(SO_4_)_2_, respectively, revealing a reduction in particle size post chromium doping. The connection between differential capacity and voltage is illustrated in Figure 8e,f, where the compound NaFe_0.8_Cr_0.2_(SO_4_)_2_ demonstrates diminished electrochemical polarization during the charge and discharge processes, meaning that Cr doping enhances the electrochemical stability of NFSO, but the capacity is somewhat reduced, as also expressed in Figure 8g [105]. Another approach is the hetero-valent replacement at non-metal sites, which can break Fe-O bonds, and the hetero-valent replacement mechanism introduces charge exchange pathways in the anionic groups, allowing polyanionic groups to conduct electrons, thereby improving the material’s intrinsic conductivity. However, there is currently less research in this area. F-doped sulfates (0 ≤ x ≤ 1) are considered stable cathodes for SIBs [97,106,107]. Incorporating experimental evidence with Density Functional Theory (DFT) computations has demonstrated that fluorine (F) doping can alter the oxidation state of specific local elements, resulting in geometric distortions of the neighboring polyhedral structures, thereby reducing the bandgap and accelerating the diffusion of Na^+^. For instance, Prabeer Barpanda and colleagues synthesized NaFeSO_4_F using low-temperature solid-state and ionothermal methods. Figure 8h shows the XRD and structural model of NaFeSO_4_F. Figure 8i,j shows SEM and TEM images of NaFeSO_4_F, respectively, showing that metal fluorosulfates generally exist stably up to 325 °C and exhibit a significant increase in conductivity [108]. M. Gnanavel and others synthesized a hydroxy NFSO—Na_3_Fe_3_(SO_4_)_2_(OH)_6_, and Figure 8k is a TEM image of Na_3_Fe_3_(SO_4_)_2_(OH)_6_. This material has stable cycling performance and high specific capacity, providing some reference significance for the non-metal doping of NFSO [109,110].

In summary, elemental doping/substitution can adjust the electronic structure and enhance the intrinsic electronic conductivity of the material, thereby improving its electrochemical performance and providing greater potential for its commercialization in sodium-ion batteries.

Finally, the advantages of three modification methods, namely nano engineering, hierarchical structure design, and element doping, were summarized, as shown in Table 1:

#### 3.3.3. Modification of Electrolytes

**Figure 9:**
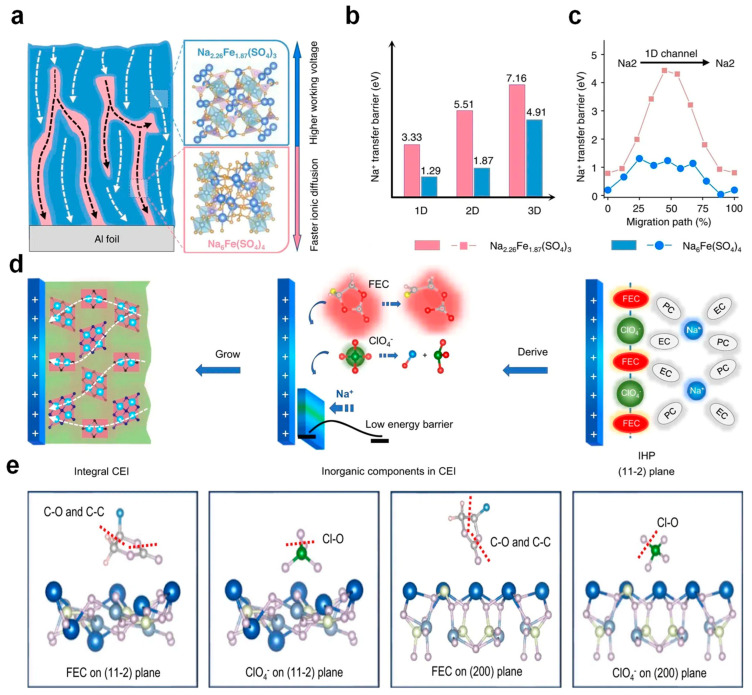
(**a**) A diagrammatic representation of the heterostructure is provided, where the blue and red areas denote Na_2.26_Fe_1.87_(SO_4_)_3_ and Na_6_Fe(SO_4_)_4_ in the cathode material, respectively. The white and black arrows illustrate the pathways of sodium-ion transport within Na_2.26_Fe_1.87_(SO_4_)_3_ and Na_6_Fe(SO_4_)_4_. Additionally, the blue and red arrows highlight the distinctive features of Na_2.26_Fe_1.87_(SO_4_)_3_, which operates at a higher voltage, and Na_6_Fe(SO_4_)_4_, which facilitates faster ionic diffusion. (**b**) The migration barriers for sodium ions are depicted across various directions, indicating the ease or difficulty of ion movement. (**c**) The energy profiles along the path Na2–Na2 in a one-dimensional direction are presented, which can reflect the energy changes sodium ions experience during migration. (**d**) A schematic diagram of interfacial adsorption and the formation of the CEI is shown. The blue flat column symbolizes the active material of the cathode, while the balls on the right represent ions and solvent molecules from the electrolyte. The small flat panel in the middle signifies the CEI formed by electrolyte decomposition. The green and red areas on the left indicate organic and inorganic components within the CEI, respectively. The white dashed lines represent the sodium-ion transport pathways within the CEI. (**e**) Calculated models are depicted, where blue, dark gray, light yellow, lilac, gray, light pink, green, and water balls correspond to Na, Fe, S, O, C, H, Cl, and F atoms, respectively. The sticks between atoms represent chemical bonds, and the red dotted line indicates the positions where these bonds are broken. Ref. [114] Copyright 2023 Springer Nature.

Surface modification and structural modification strategies are common methods to improve interface stability and maintain structural integrity during the cycling of high-voltage cathodes. However, it must be noted that a battery is an integrated system, and other components such as electrolytes, conductive carbon, and binders also play a key role in determining recyclability [111,112,113]. The modification of electrolytes primarily revolves around the utilization of fluorinated electrolytes, characterized by high-lying unoccupied molecular orbitals (LUMO) and low-lying highest occupied molecular orbitals (HOMO). This characteristic implies that the electrolyte is more inclined to form a solid electrolyte interphase (SEI) on the anode’s surface and is relatively resistant to oxidation at the cathode side. In addition, fluorinated electrolytes also have characteristics such as low melting points and high thermal stability. Zhang and the team created a sodium-ion conductive, high-voltage cathode material, Na_2.26_Fe_1.87_(SO_4_)_3_, which incorporates a Na_6_Fe(SO_4_)_4_ phase heterostructure and an electronically conductive carbon network (denoted as NFSO-H). This synthesis was achieved through a co-precipitation method, succeeded by a calcination process. Figure 9a is a schematic diagram of the heterostructure. Figure 9b,c show the migration energy barriers for Na^+^ along different directions and the energy distribution along the one-dimensional path Na2–Na2, respectively. Researchers have discovered that the dynamics of ion transfer are intricately linked to the interface structure and the composition within the bulk material. A cathode surface that is highly reactive can result in reduced ionic conductivity and the formation of a substantial CEI (Cathode Electrolyte Interphase). This can impede the rapid and reversible movement of Na^+^ ions at the interface, as depicted in Figure 9d. Following this observation, a computational model was developed to simulate the adsorption and decomposition behavior of electrolyte components, such as FEC (fluoroethylene carbonate) and ClO_4_^−^ (perchlorate ions), on various cathode crystal facets. This model was utilized to calculate the energy required for bond dissociation, as illustrated in Figure 9e [114].

In summary, the use of fluorinated electrolytes can form a highly stable CEI layer while reducing side effects caused by oxidation and reduction reactions. Moreover, fluorinated electrolytes feature a low melting point and high thermal stability, making them more suitable for high-voltage conditions. This plays a crucial role in improving the performance and safety of sodium-ion batteries, particularly in supporting high-voltage and high-energy-density cathode materials [57,73,114,115,116].

**Figure 10:**
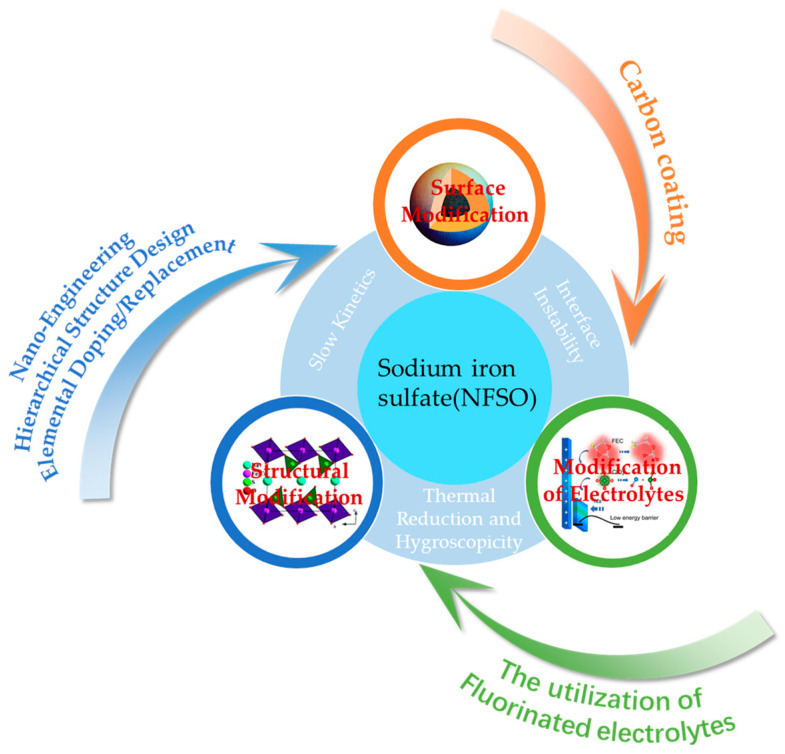
An overview diagram of this article.

SIBs, known for their rapid charge-discharge capabilities and economical nature, present extensive potential for use in energy storage applications. The cathode material, serving as the origin of sodium ions within the battery, has a direct influence on the battery’s rate performance due to its kinetic characteristics. This paper selects the NFSO cathode material, which has excellent stability and safety, as the subject of discussion. Figure 10 is an overview diagram of this article. The electrochemical performance of NFSO synthesized by different methods is shown in Table 2. It first explores the intrinsic factors that determine the kinetic performance of the material, as well as the material’s structure. When selecting and designing the material structure, the goal should be to create multi-dimensional diffusion channels. However, materials with low-dimensional or no diffusion channels can also improve kinetic performance by creating diffusion pathways through different methods, such as composites with carbon nanotubes. The paper then reviews the current strategies for improving the kinetic performance of NFSO cathode materials, such as enhancing rate performance from the perspective of the overall morphology of the electrode material through surface modification, nano-engineering, and hierarchical structure design, or improving the material’s inherent electrical conductivity through elemental doping or substitution. The current analysis of inherent properties is insufficient, and future work should provide more fundamental and scientific explanations from the perspective of the material itself for the diffusion mechanism of sodium ions or the action mechanism of additives. Since SIBs and LIBs have similar working principles, some means of enhancing the performance of LIB electrodes are relatively mature and worth emulating [66,67,68,69]. Additionally, optimization strategies should be specifically analyzed for each material, and specific optimization should be carried out according to the characteristics of the material to avoid blindly selecting improvement plans [42]. In the future, sodium-ion batteries will be widely used in transportation, flexible electronics, and energy storage [25,117,118,119,120,121,122,123,124,125,126,127].

## 4. Summary and Outlook

**Figure 11:**
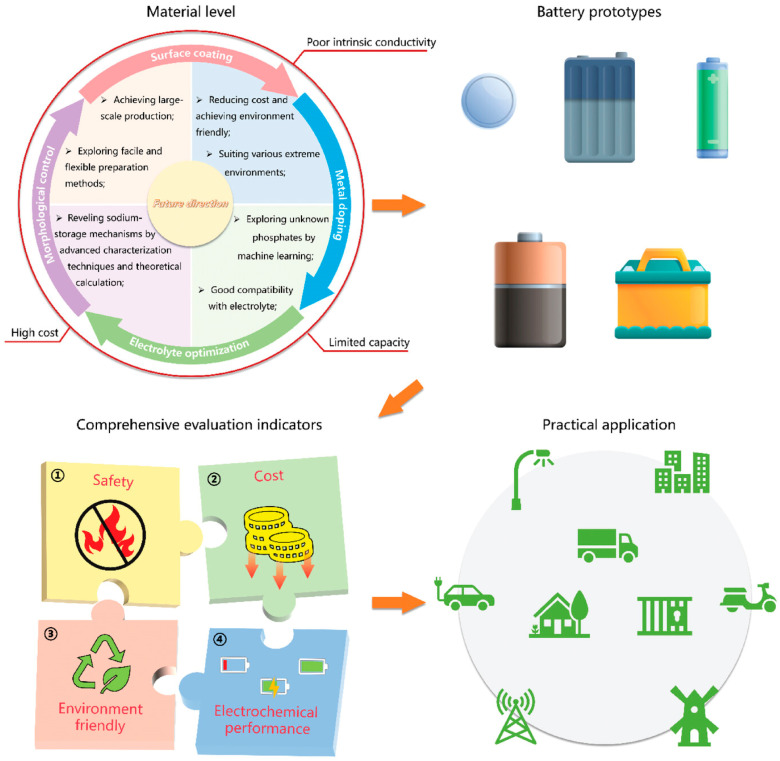
The future direction of phosphate-based polyanionic cathodes for SIBs toward commercialization. Ref. [42] Copyright 2023 Wiley-VCH Verlag.

This paper mainly summarizes the structure, sodium storage mechanism, advantages and disadvantages, and modification methods of SIBs and the polyanionic sulfate-NFSO. It offers valuable insights for enhancing the electrochemical performance of these materials in SIBs and also aids in the development of innovative SIB cathode materials. It is possible to replace the transition metal elements in them to study new SIB cathode materials (Figure 11). Although sodium iron sulfate (NFSO) as a cathode material for sodium-ion batteries exhibits numerous advantages, such as a high voltage platform, excellent cycling stability, and low cost, it still faces several challenges in practical applications. Future research should focus on the following directions: The inherently low electronic conductivity of NFSO limits its rapid charge and discharge performance. Advanced doping strategies, interface engineering, and nanostructure design can be employed to enhance the material’s electronic and ionic transport properties. Additionally, combining high-precision computational modeling with experimental studies can help uncover the microscopic mechanisms of ion diffusion pathways in NFSO, providing a more scientific basis for modification strategies. For high-voltage cathode materials, more stable electrolytes, such as those based on novel fluorides or borates, should be developed to reduce side reactions and enhance interface stability. Research into multifunctional electrolyte additives could not only stabilize the CEI/SEI interface but also improve sodium-ion transport efficiency. Efforts should be made to advance the application of NFSO-based sodium-ion batteries in energy storage systems, such as renewable energy storage and grid load balancing. For electric vehicles, the development of compact, high-energy-density sodium-ion battery modules could support green transportation. In the future, through interdisciplinary collaboration and international cooperation, combining theoretical guidance with experimental validation, it is expected that the bottlenecks of NFSO in practical applications can be overcome, enabling it to become a representative of high-performance, low-cost cathode materials for sodium-ion batteries and contributing to innovations in energy storage technology.

## Figures and Tables

**Table 1:** Comparison of the advantages of different modification methods.

Methods	Advantage		
Nano-Engineering	Nanowires/nanorods	Increase the surface area of the material and accelerate the transfer rate of electrons and ions at the interface; Provide more reactive sites to enhance electrochemical activity; Micro nano structures counteract the aggregation of NFSO	
Hierarchical Structure Design	One dimensional nanostructure	It shortens the diffusion distance of Na+	
	Two-dimensional nanostructures	It can be made into 3D mesh structures, which helps enhance structural stability	
	Three-dimensional nanostructure	Like a nano flower-shaped hierarchical structure, it enhances the diffusion of sodium ions and the wettability of electrolytes, improving rate performance	
Elemental Doping/Replacement	General doping	Improve intrinsic conductivity and enhance electrochemical performance	
	Special doping	Doping Mn/Al	Improve structural and thermal stability
		Doping Co	Increased reaction kinetics
		Doping Mg/Zr/Ti	Adjust conductivity and lifespan

**Table 2:** Comparison of Electrochemical Performance of NFSO Synthesized by Different Methods.

Materials	Preparation Method	Cyclic Performance	Ref.
Na_2_Fe(SO_4_)_2_	low-temperature solid-state method	50 mA h g^−1^ and 83 mA h g^−1^ at 0.5 C at 0 °C and 55 °C	[57]
	co-precipitation and calcination	80.69 and 65.32% capacity retention after 1300 cycles at 60 mA g^−1^ and 5700 cycles at 600 mA g^−1^	[114]
	liquid phase method	discharge capacity remains 71% of initial capacity after 100 cycles at 1 C	[115]
Na_2_Fe(SO_4_)_2_@rGO/C	facile homogeneous strategy	high voltage plateau of 3.75 V, 85 mAh g^−1,^ and 330 Wh kg^−1^ at 0.05 C	[73]
Na_6–2*x*_Fe*_x_*(SO_4_)_3_	solid-phase ball-milling method	104.5 mAh g^−1^ at 0.1 C	[116]

## Data Availability

No new data were created or analyzed in this study.
